# Diuretic and Natriuretic Effects of Hesperidin, a Flavanone Glycoside, in Female and Male Hypertensive Rats

**DOI:** 10.3390/plants12010025

**Published:** 2022-12-21

**Authors:** Priscila de Souza, Rita de Cássia Vilhena da Silva, Luisa Nathália Bolda Mariano, Sabrina Lucietti Dick, Giovana Cardozo Ventura, Valdir Cechinel-Filho

**Affiliations:** Postgraduate Program in Pharmaceutical Sciences, University of Vale do Itajaí (UNIVALI), Rua Uruguai, 458, Centro, Itajaí 88302-901, Brazil

**Keywords:** diuresis, natriuresis, cholinergic, flavanone

## Abstract

Hesperidin (HSP) is a major flavanone glycoside in citrus fruits, including sweet oranges and lemons. It demonstrates numerous pharmacological activities, such as antihypertensive effects and cardiac and kidney tissue protection. However, its effect on modulating renal function has yet to be properly explored. Female and male Wistar spontaneously hypertensive rats (SHR) were used to test the effect of HSP on renal function. The rats were divided into different groups, treated orally, and placed in metabolic cages for urine collection for 8 h. HSP, at doses of 0.3–3 mg/kg, led to an increase in urine volume in both female and male SHR. This effect was associated with increased Na^+^ elimination (3 mg/kg) without causing any change in K^+^ excretion or pH and conductivity values. When given HSP in combination with hydrochlorothiazide (HCTZ) or amiloride (AMLR), urine volume and Na^+^ elimination were significantly increased compared to the group that received only HSP. In relation to K^+^ excretion, the depleting effect of HCTZ and the sparing of AMLR prevailed in both groups. Pre-treatment with a non-selective cholinergic receptor antagonist, atropine, partially prevented HSP-induced diuresis and natriuresis in male SHR, but this effect was not demonstrated with the non-selective inhibitor of the enzyme cyclooxygenase, indomethacin. This study shows the diuretic action of HSP in hypertensive rats, an activity probably associated with the cholinergic pathway. Although various biological actions have already been defined for HSP, this pioneering research reveals its potential as a diuretic medicine.

## 1. Introduction

Cardiovascular diseases (CVD) are the leading cause of death globally, and hypertension is one of the most prevalent CVD [1]. Pharmacological therapy should be initiated at a systolic blood pressure (SBP) higher than or equal to 140 mm Hg and/or diastolic blood pressure (DBP) higher than or equal to 90 mm Hg [2]. One of the drug classes used is diuretics, considered the first-line option and effective in lowering blood pressure in hypertensive individuals [3]. Diuretics are also effective in many other disorders, including hepatic cirrhosis, acute and chronic kidney failure, nephrotic syndrome, and congestive heart failure, thus reducing the likelihood of a cardiovascular event [4].

Despite the availability of various therapies to lower blood pressure (BP), control of BP is not achieved in many patients. Along with traditional pharmacological treatments, alternative treatments, such as natural-based products, are used to prevent cardiovascular diseases such as hypertension, diabetes, and obesity [5]. Plants produce secondary metabolites that have several functions. Among them, we find a large class of polyphenols. The biological activities of polyphenols in preventing and treating various pathologies are already well described in the literature [6]. The most important classes of polyphenols are flavonoids, which include flavanols (found in tea, for example), flavanones (in citrus fruits, for example), flavonols (in tea, apple, and onions for example), and nonflavonoids (found in wine, for example), and anthocyanins (in cherry, for example) [7]. Citrus fruits contain flavonoid compounds, including hesperidin, hesperetin, naringin, naringenin, diosmin, and others [8]. Hesperidin represents more than 90% of the flavonoids in sweet oranges; among their biological activities, citrus flavonoids possess antioxidant effects through direct radical scavenging and anti-inflammatory and hypoglycemic properties. They have the ability to improve several pathologies, such as cancer and cardiovascular diseases [9]. Several authors have demonstrated that oral administration of hesperidin reduces systolic blood pressure in spontaneously hypertensive rats. This fact is due to the increase in nitric oxide production and improvement in vascular function [10]. Hesperidin also reduces blood pressure, in a dose-dependent manner, by lowering the renin-angiotensin system (RAS) activity. It also has an antioxidant effect in rats subjected to the two-kidney, one-clip (2K-1C) model [11].

Considering the results mentioned above, and despite the broad scale of pharmacologic activities and widespread application of hesperidin, we have yet to find any studies demonstrating its effect on modulating renal function. Based on this information, this study evaluates the diuretic action of hesperidin in spontaneously hypertensive rats.

## 2. Results

### 2.1. Diuretic and Natriuretic Effect of HSP in Female and Male Hypertensive Rats

**Figure 1:**
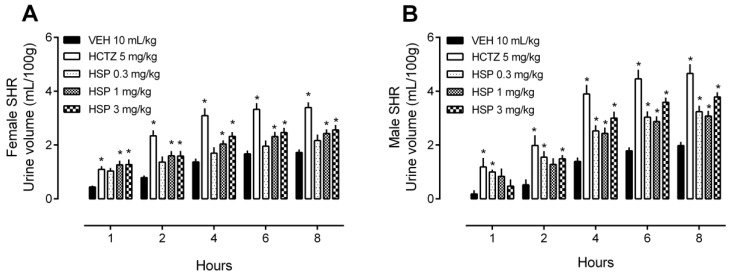
Urine volume of female and male spontaneously hypertensive rats (SHR) treated with hesperidin (HSP). (**A**) Urine volume of female rats after treatment with different doses (0.3–3 mg/kg) of HSP. (**B**) Urine volume of male rats after treatment with different doses (0.3–3 mg/kg) of HSP. The values show the mean ± SEM. Statistical analysis was performed by means of two-way ANOVA followed by Dunnett’s multiple comparisons test. * *p* < 0.05 when compared with the VEH (vehicle; saline 10 mL/kg). HCTZ = hydrochlorothiazide.

Preliminary testing for biological activity was initially carried out to determine the capacity of HSP to modify renal function (i.e., urine volume and electrolyte excretion) in hypertensive female and male rats. As shown in Figure 1A, HSP at doses of 1 and 3 mg/kg, but not 0.3 mg/kg, significantly increased the urinary volume when given orally to female SHR. Moreover, as shown in Figure 1B, HSP at doses of both 0.3, 1, and 3 mg/kg significantly increased the urine volume when given orally to male SHR. Clinical standard thiazide-type diuretic hydrochlorothiazide (HCTZ) was used as an internal control of the tests, and as expected, it changed the volume of urine and the other urinary parameters already known in the clinical practice.

**Figure 2:**
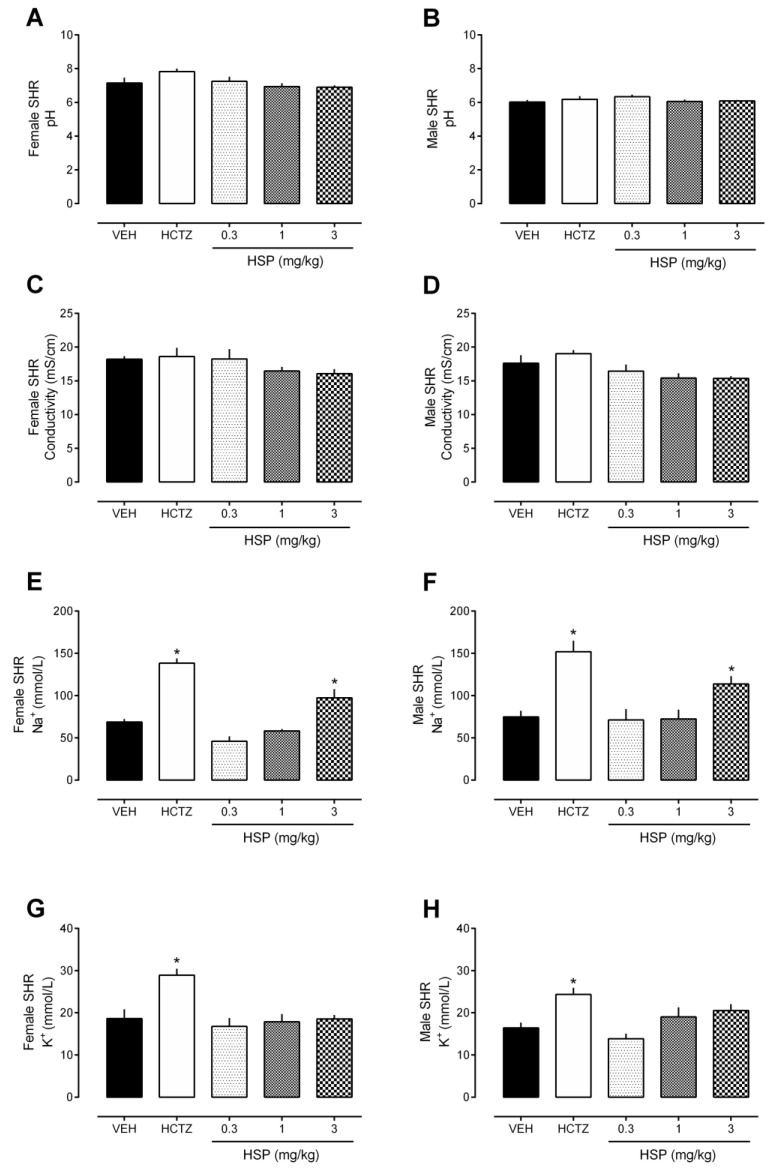
Urinary parameters (8 h) after oral treatment with hesperidin (HSP). (**A**,**B**) Urine pH values. (**C**,**D**) Urine conductivity values. (**E**,**F**) Urine Na^+^ excretion. (**G**,**H**) Urine K^+^ excretion. The values show the mean ± SEM. Statistical analysis was performed by means of one-way ANOVA followed by Dunnett’s multiple comparisons test. * *p* < 0.05 when compared with the VEH (vehicle; saline 10 mL/kg). HCTZ = hydrochlorothiazide.

Regarding urine pH and conductivity values, no significant changes were found after the treatments (Figure 2A–D). The same occurred during all the tests presented in the sequence (data not shown).

As shown in Figure 2E,F, treatment with HSP (3 mg/kg) also increased urinary Na^+^ excretion, without altering the levels of K^+^ (Figure 2G,H), in both female and male SHR.

### 2.2. HSP-Induced Diuresis and Natriuresis and Its Combination with Diuretic Drugs

**Figure 3:**
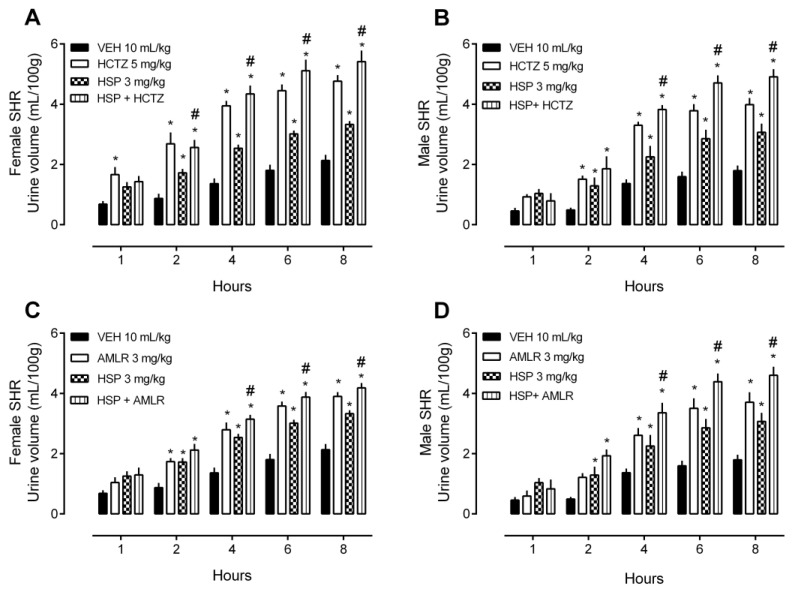
Urine volume of female and male spontaneously hypertensive rats (SHR) treated with hesperidin (HSP) in combination with hydrochlorothiazide (HCTZ) or amiloride (AMLR). (**A**) Urine volume of female rats after treatment with HSP plus HCTZ. (**B**) Urine volume of male rats after treatment with HSP plus HCTZ. (**C**) Urine volume of female rats after treatment with HSP plus AMLR. (**D**) Urine volume of male rats after treatment with HSP plus AMLR. The values show the mean ± SEM. Statistical analysis was performed by means of two-way ANOVA followed by Dunnett’s multiple comparisons test. * *p* < 0.05 when compared with the VEH (vehicle; saline 10 mL/kg); # *p* < 0.05 when compared with the HSP only group.

**Figure 4:**
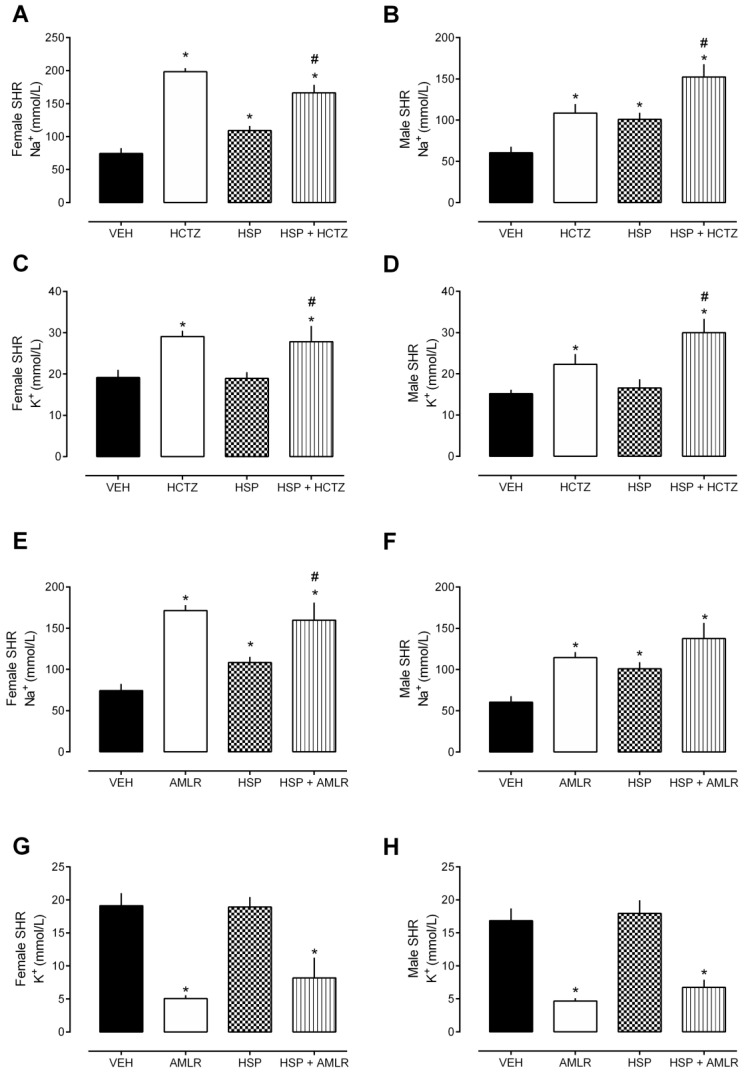
Urinary parameters (8 h) after oral treatment with hesperidin (HSP) in combination with hydrochlorothiazide (HCTZ) or amiloride (AMLR). (**A**,**B**) Urine Na^+^ excretion after treatment with HSP plus HCTZ. (**C**,**D**) Urine K^+^ excretion after treatment with HSP plus HCTZ. (**E**,**F**) Urine Na^+^ excretion after treatment with HSP plus AMLR. (**G**,**H**) Urine K^+^ excretion after treatment with HSP plus AMLR. The values show the mean ± SEM. Statistical analysis was performed by means of one-way ANOVA followed by Dunnett’s multiple comparisons test. * *p* < 0.05 when compared with the VEH (vehicle; saline 10 mL/kg); # *p* < 0.05 when compared with the HSP-only group.

When the rats were given HSP (3 mg/kg) in combination with HCTZ (5 mg/kg), the urine volume (Figure 3A,B) and Na^+^ elimination (Figure 4A,B) were significantly enhanced when compared to the group that received only HSP. These results were similar in female and male SHR. As HCTZ classically increases the loss of K^+^ in the urine, with the combination of HSP plus HCTZ, the effect of HCTZ prevailed (Figure 4C,D).

In addition, when given HSP (3 mg/kg) in combination with AMLR (3 mg/kg), the urine volume (Figure 3C,D) and Na^+^ elimination (Figure 4E,F) were significantly enhanced when compared to the group that received only HSP. We also detected the classic effect of AMLR in sparing K^+^ excretion, and this effect prevailed in combination with HSP (Figure 4G,H).

### 2.3. Mechanisms Involved in the Diuretic Action Stimulated by HSP

**Figure 5:**
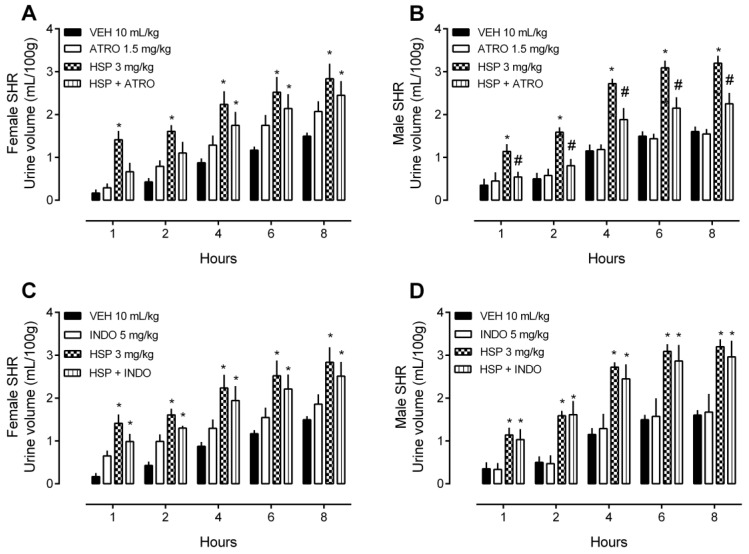
Urine volume of female and male spontaneously hypertensive rats (SHR) treated with hesperidin (HSP) in the presence of atropine (ATRO) or indomethacin (INDO). (**A**) Urine volume of female rats after treatment with HSP in the presence of ATRO. (**B**) Urine volume of male rats after treatment with HSP in the presence of ATRO. (**C**) Urine volume of female rats after treatment with HSP in the presence of INDO. (**D**) Urine volume of male rats after treatment with HSP in the presence of INDO. The values show the mean ± SEM. Statistical analysis was performed by means of two-way ANOVA followed by Dunnett’s multiple comparisons test. * *p* < 0.05 when compared with the VEH (vehicle; saline 10 mL/kg); # *p* < 0.05 when compared with the HSP-only group.

**Figure 6:**
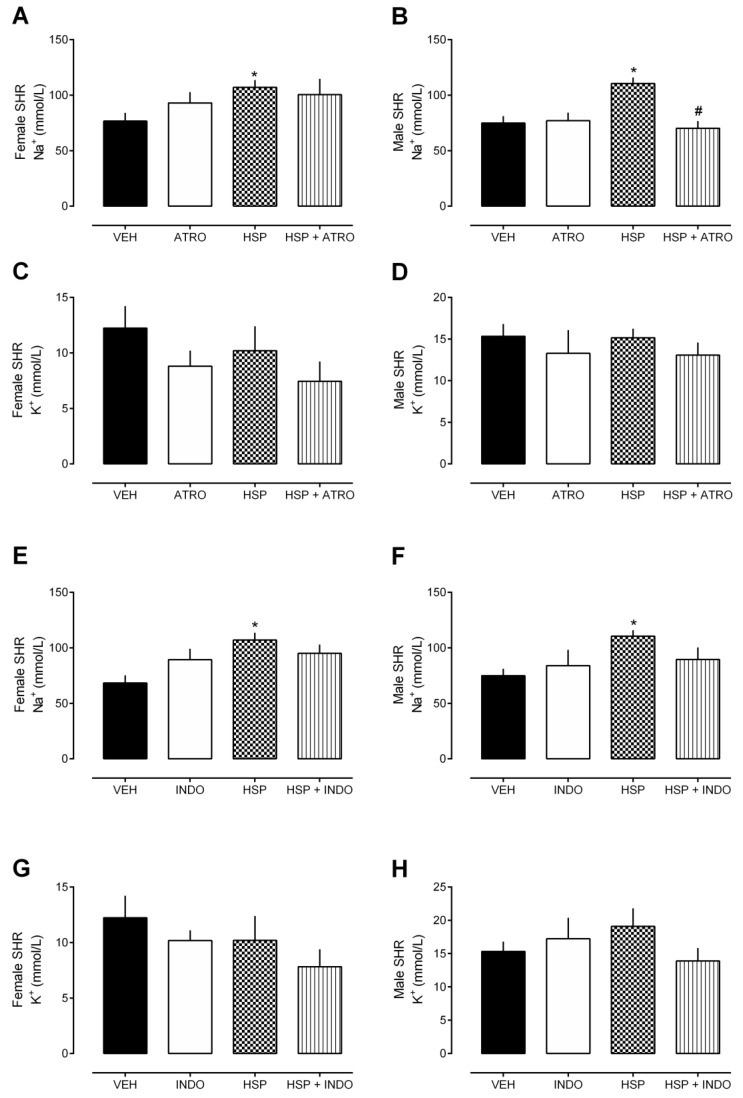
Urinary parameters (8 h) after oral treatment with hesperidin (HSP) in the presence of atropine (ATRO) or indomethacin (INDO). (**A**,**B**) Urine Na^+^ excretion after treatment with HSP in the presence of ATRO. (**C**,**D**) Urine K^+^ excretion after treatment with HSP in the presence of ATRO. (**E**,**F**) Urine Na^+^ excretion after treatment with HSP in the presence of INDO. (**G**,**H**) Urine K^+^ excretion after treatment with HSP in the presence of INDO. The values show the mean ± SEM. Statistical analysis was performed by means of one-way ANOVA followed by Dunnett’s multiple comparisons test. * *p* < 0.05 when compared with the VEH (vehicle; saline 10 mL/kg); # *p* < 0.05 when compared with the HSP-only group.

Separate groups of rats were pre-treated with atropine or indomethacin and received HSP or only vehicle (i.e., saline 10 mL/kg) after. The non-selective cholinergic receptor antagonist, ATRO, but not the non-selective inhibitor of the enzyme cyclooxygenase, INDO, partially prevented HSP-induced diuresis (Figure 5) and natriuresis (Figure 6) in male SHR. Notably, both ATRO (1.5 mg/kg) and INDO (5 mg/kg), at the doses used, were incapable of causing any change in renal function, as shown in Figure 5 and Figure 6.

## 3. Discussion

There is an important benefit in secondary active ingredients acquired from plant species for treating diseases in humans, such as those affecting the circulatory system and kidneys. Meanwhile, the ability to modulate the hydro electrolyte balance makes diuretic drugs helpful in treating a range of ailments, including heart failure, hypertension, hepatic cirrhosis, nephrotic syndrome, nephrogenic diabetes insipidus, and other edematous diseases [3,4]. Different compounds isolated from plants have already been reported to induce diuresis in experimental studies [12,13]. However, despite the various pharmacological activities already described for hesperidin, this is the first report of its possible diuretic effect. In fact, hesperidin has a long history of consumption and is known as vitamin P. Hesperidin (C_28_H_34_O_15_) is chemically classified as a flavanone glycoside containing hesperetin (the aglycone form) and sucrose [14]. In clinical practice, a mixture containing 90% diosmin and 10% flavonoids expressed as hesperidin is commercialized as a vasoprotective venotonic agent, normalizing capillary permeability and reinforcing capillary resistance in the microcirculation and increasing lymphatic drainage. All these actions lead to an improvement in symptoms related to chronic venous insufficiency of the lower limbs. Due to its clinical application, there is much evidence of the low toxicity and safety of its use, given the absence of toxicity and uninfluenced systemic parameters [15,16].

The findings revealed herein demonstrated that hypertensive rats orally treated with hesperidin presented a significant increase in urinary volume and Na^+^ elimination when compared to the vehicle (saline 10 mL/kg)-only group. These data were similar to the values achieved with hydrochlorothiazide, a thiazide-class diuretic agent used as a control. On the other hand, unlike hydrochlorothiazide, hesperidin did not cause any change in K+ excretion. This is an important difference since many adverse effects are related to excessive loss of K+ in the urine. Based on these data, we can suggest that hesperidin acts through a different mode of action from classic diuretics.

Another important point of this study is that all the experiments were conducted using both female and male animals. Most experimental animal studies use only males and exclude females, but this can result in a loss of understanding of illness processes in females. It also results in fewer opportunities to study female-individual incidents, such as hormonal protection against systemic arterial hypertension [17]. Considering the importance of demonstrating efficacy in both sexes, this study revealed that hesperidin has similar effects when given to both females and males, ruling out any doubt in the understanding of this specific effect. Furthermore, this study was entirely conducted in hypertensive animals. Considering two other important points, first, it studies a biological effect in an appropriate disease model, and second, it uses spontaneously hypertensive rats (SHR), which are the most commonly used animal model of essential hypertension [18]. As in humans, hemodynamic and metabolic disorders in SHR are also revealed by multifactorial pathways. Briefly, the hypertensive injury in SHR is pressure-related, with the vascular impairment causing arterial hypertrophy in the juxtamedullary cortex of kidney tissue. The progression of arteriolar hypertrophy, in turn, leads to the failure of some glomerular mesangial regions and tubular atrophy, leading to reduced glomerular filtration [19]. Considering all the renal changes evidenced in the SHR, this model represents an excellent experimental tool to delineate strategies aimed at tissue protection and restoration of functional and hemodynamic parameters. In this sense, hesperidin has potential application for future studies, as it was able to significantly alter renal function in SHR.

In clinical practice, treatment using two diuretics of different classes is very common, aimed at increasing efficacy and reducing side effects. It is known that thiazide and loop diuretics have greater diuretic power, but both cause excessive K^+^ loss. On the other hand, K^+^-sparing diuretics, despite the benefit of this electrolyte, have low diuretic power. Therefore, combining these classes is more appropriate for clinical treatment, as it combines diuretic power with greater control of electrolyte excretion [20]. The combination of hesperidin with hydrochlorothiazide (which acts on the distal tubule of the nephron, interrelating directly with the electrolytic reabsorption apparatus and increasing the excretion of Na^+^, K^+^, and Cl^−^, thereby increasing the volume of liquid loss [21]) or with amiloride (which acts on the distal tubule of the nephron, blocking Na+ transport, and inhibiting Na^+^- K^+^ exchange [22]) was very positive since the diuretic and natriuretic actions were significantly enhanced, with the effects of each class prevailing under the elimination of K^+^. These combinations can therefore be used to treat hyperkalemia or hypokalemia, respectively.

Seeking to suggest a possible mode of action of hesperidin in including diuresis, we carried out experiments to determine the involvement of cholinergic receptors endogenously activated by the acetylcholine and cyclooxygenase pathways with the generation of vascular-relaxing endoperoxides. The production of vasodilator intermediaries in the kidneys improves the glomerular filtration level by opposing the constrictor agents [23,24]. The non-selective cholinergic receptor antagonist atropine, but not the non-selective inhibitor of the enzyme cyclooxygenase indomethacin, partially prevented hesperidin-induced diuresis and natriuresis in male SHR, at least in part, the activation of cholinergic receptors for the renal effects of hesperidin. Some previous studies have already reported the biological effects attributed to hesperidin as a possible modulation of the cholinergic pathway [10,25]. In addition, a recent study identified hesperidin as a type three muscarinic receptor (M_3_R) ligand [26]. Although we cannot discuss receptor activation or blockade, there is, at least, evidence of the interaction of hesperidin with this target. Finally, a study also shows that hesperidin can inhibit the carbonic anhydrase enzyme in rats’ testis, liver, and heart tissues [27]. Carbonic anhydrase inhibitors are used in the clinic to treat glaucoma and as a diuretic agent. However, based on the results of the present study, and considering that treatment with hesperidin did not harm the urinary pH values, we can suggest that the diuretic effect presented may be unrelated to the inhibition of this enzyme. Further studies are needed to investigate the pathways involved in the pharmacological effects described here.

## 4. Materials and Methods

### 4.1. Drugs and Reagents

Amiloride, atropine, hesperidin, hydrochlorothiazide, and indomethacin were obtained from Sigma-Aldrich Chemical Co. (St. Louis, MO, USA). The other substances were acquired from Merck (Darmstadt, Germany).

### 4.2. Animals

In this study, we used male and female spontaneously hypertensive rats (SHR) (aged 12 to 16 weeks) provided by the University of Vale do Itajaí after obtaining the approval of the institutional ethics committee (authorization no. 028/20p). The animals were maintained in a regular environment, with a 12 h light/dark cycle and temperature of 22 ± 2 °C, and with free access to food and water.

### 4.3. Assessment of Diuresis and Analytical Procedures

For these experiments, all the animals were fasted for 8 h, with unrestricted availability of water. The rats were randomly distributed into groups of 6 animals each and were given oral physiological saline (NaCl 0.9%, 5 mL/100 g) to induce uniform diuresis in all the animals. Male and female SHR were orally treated with the vehicle (VEH; saline 10 mL/kg), hydrochlorothiazide (HCTZ; 5 mg/kg), or hesperidin (HSP; 0.3, 1, and 3 mg/kg). Once treated, the animals were allocated to individual metabolic cages, and the urine was collected in the first hour and every 2 h, with the final collection at 8 h. The cumulative urinary output was expressed per 100 g body weight. The pH values, conductivity, and electrolytes excretion (Na^+^, K^+^), were measured in each urine sample at the end of the experiment. For details on the analytical procedures, see the reference list [28,29,30].

In another set of experiments, we evaluated the effect of HSP in association with other diuretics and investigated the possible mechanism of action using pharmacological tools. For this, rats were treated with HCTZ (5 mg/kg), amiloride (3 mg/kg), atropine (1.5 mg/kg), and indomethacin (5 mg/kg) alone and in combination with HSP (3 mg/kg). Immediately after the treatments, the rats were kept in metabolic cages under the conditions described above.

### 4.4. Statistical Analysis

Statistical analysis was performed by one-or-two way of analysis of variance (ANOVA) followed by Dunnett’s multiple comparison test using GraphPad Prisma version 6.00 for Windows (GraphPad Software, La Jolla, CA, USA). All the data are presented as mean ± standard error of the mean (S.E.M.) of 6 animals per group, and a *p*-value less than 0.05 was considered statistically significant.

## Data Availability

The datasets generated and/or analyzed as part of the current study are available from the corresponding author upon request.
